# Roles of Clonal Integration in both Heterogeneous and Homogeneous Habitats

**DOI:** 10.3389/fpls.2016.00551

**Published:** 2016-04-26

**Authors:** Haijie Zhang, Fenghong Liu, Renqing Wang, Jian Liu

**Affiliations:** ^1^Institute of Environmental Research, Shandong UniversityJinan, China; ^2^National Science Library, Chinese Academy of SciencesBeijing, China; ^3^School of Life Sciences, Shandong UniversityJinan, China

**Keywords:** *Alternanthera philoxeroides*, clonal integration, heterogeneous habitat, homogeneous habitat, nutrients, severing

## Abstract

Many studies have shown that clonal integration can promote the performance of clonal plants in heterogeneous habitats, but the roles of clonal integration in both heterogeneous and homogeneous habitats were rarely studied simultaneously. Ramet pairs of *Alternanthera philoxeroides* (Mart.) Griseb were placed in two habitats either heterogeneous or homogeneous in soil nutrient availability, with stolon connections left intact or severed. Total biomass, total length of stolons, and number of new ramets of distal (relatively young) ramets located in low-nutrient environments were significantly greater when the distal ramets were connected to than when they were disconnected from proximal (relatively old) ramets located in high-nutrient environments. Total length of stolons of proximal ramets growing in low-nutrient environments was significantly higher when the proximal ramets were connected to than when they were disconnected from the distal ramets growing in high-nutrient environments, but stolon connection did not affect total biomass or number of new ramets of the proximal ramets. Stolon severing also did not affect the growth of the whole ramet pairs in heterogeneous environments. In homogeneous high-nutrient environments stolon severing promoted the growth of the proximal ramets and the ramet pairs, but in homogeneous low-nutrient environments it did not affect the growth of the proximal or distal ramets. Hence, for *A. philoxeroides*, clonal fragmentation appears to be more advantageous than clonal integration in resource-rich homogeneous habitats, and clonal integration becomes beneficial in heterogeneous habitats. Our study contributes to revealing roles of clonal integration in both heterogeneous and homogeneous habitats and expansion patterns of invasive clonal plants such as *A. philoxeroides* in multifarious habitats.

## Introduction

Plant invasion has become a significant threat to biodiversity, environment, and economy both globally and locally (Mack et al., 2000; Liu et al., 2005; Vila et al., 2011). Many notorious invasive plants are clonal, with the capability of vigorous clonal propagation (Dong, 1996; Pysek et al., 2003; Liu et al., 2006; Wang et al., 2008; Keser et al., 2014). For example, *Alternanthera philoxeroides* (Mart.) Griseb is an invasive clonal plant, which has heavily invaded many areas of the world (Julien et al., 1995; Ye et al., 2003; Wang et al., 2008; Zhang et al., 2015). Clonal growth has been considered an important trait for invasive clonal plants (Liu et al., 2008; Wang et al., 2009; Xu et al., 2010; Roiloa et al., 2013; Song et al., 2013; You et al., 2013). For instance, *A. philoxeroides* can expand from terrestrial to aquatic environments with the support of clonal integration (Wang et al., 2009). Clonal integration can also aid the spreading of *A. philoxeroides* and *Vallisneria spiralis* L. into competitive environments (Xiao et al., 2011; You et al., 2014).

Heterogeneity is common in nature (Hutchings and Wijesinghe, 1997; Alpert, 1999; Dong et al., 2015; Keser et al., 2015). Numerous studies have investigated the strategies of clonal plants to cope with habitats with heterogeneous distributions of, e.g., nutrients, light, space, and others (Liu et al., 2008; Wang et al., 2009; Xu et al., 2010; Song et al., 2013; Roiloa et al., 2014b). These studies have shown that in heterogeneous environments, ramets exposed to stressful environments commonly perform better when they are integrated with ramets located in non-stressful conditions (Wang et al., 2008; Xu et al., 2010; Song et al., 2013; You et al., 2013; Roiloa et al., 2014a).

Natural environments can also be homogeneous at the scale of plant growth (Stuefer, 1996; Dong et al., 2015). Theoretical models (Caraco and Kelly, 1991; Alpert, 1999) have predicted that clonal integration may be disadvantageous in environments with a homogeneous supply of resources. In a recent study, however, Dong et al. (2015) developed a conceptual model showing that clonal integration may also have a positive effect on the growth of clonal plants when connected ramets in resource-rich habitats have different uptake abilities. Some studies have tested the roles of clonal integration in homogeneous environments (e.g., Salzman and Parker, 1985; Evans and Whitney, 1992; Yu et al., 2002; He et al., 2011), but very few have detected a significant effect.

We conducted a greenhouse experiment on *A. philoxeroides* to test effects of clonal integration in both heterogeneous and homogeneous environments. We grew ramet pairs of *A. philoxeroides* in both homogeneous high and low soil nutrient conditions and heterogeneous conditions with a high and a low soil nutrient patch, with the stolon connecting the two ramets of a pair severed or left connected. We detected that clonal integration could play significant roles in both homogeneous and heterogeneous environments.

## Materials and Methods

### Plant Materials

*Alternanthera philoxeroides* is a perennial weed from the family Amaranthaceae. It is native to South America but widespread in a variety of habitats around the world (Julien et al., 1995; Geng et al., 2007; Zhang et al., 2015). In most of the introduced regions, it reproduces asexually, primarily from stem nodes and shoot fragments (Julien et al., 1995; Geng et al., 2007). Despite the extremely low genetic diversity of *A. philoxeroides* in China (Xu et al., 2003; Wang et al., 2005), the species experienced massive vegetative propagation and rapid expansion in China (Xu et al., 2003; Ye et al., 2003; Wang et al., 2005; Zhu et al., 2015).

We collected eight *A. philoxeroides* plants from a cropland in Kunming, China, in April, and transplanted them into a greenhouse under natural sunlight and ambient temperature for propagation. After 4 months of cultivation, we selected newly produced ramet pairs that were similar in size. To eliminate possible effects of genotype, ramet pairs in every treatment were from the eight original plants. Also, genetic diversity of *A. philoxeroides* is extremely low in China (Xu et al., 2003; Wang et al., 2005). In each ramet pair, the two ramets were connected and were rooted in two plastic pots filled with river sand. The ramets were standardized to the same size (two leaves and 2-cm-long root). Standardization was conducted once a week and repeated three times.

### Experimental Design

**FIGURE 1:**
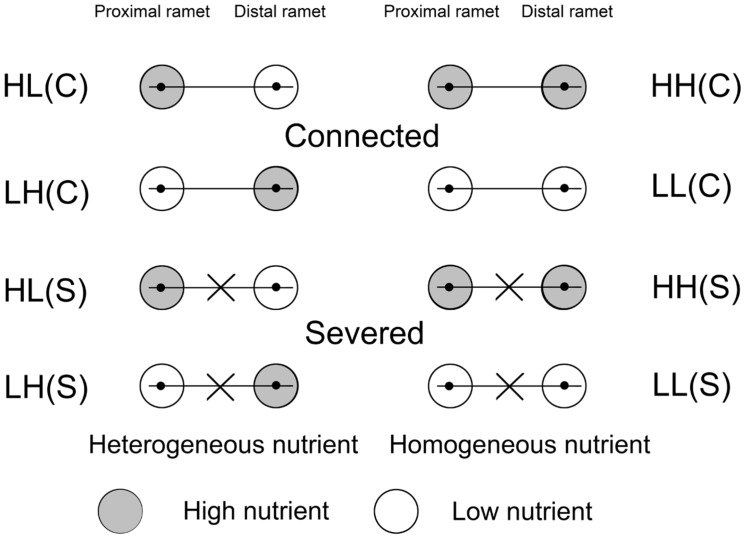
**Schematic representation of the experimental design.** Ramet pairs of *Alternanthera philoxeroides*, consisting of proximal and distal ramets, were planted in either heterogeneous or homogeneous nutrient habitats, with stolon connections left intact (C) or severed (S). Proximal and distal ramets were separately placed in two pots. The gray and white circles represented high (H) and low (L) nutrients, respectively.

The treatments with different soil nutrient availabilities and stolon severing were performed on August 14. The ramets in each pair (64 pairs) were exposed to: (1) heterogeneous distribution of soil nutrients (high in proximal ramet and low in distal ramet, HL); (2) heterogeneous distribution of soil nutrients (low in proximal ramet and high in distal ramet, LH); (3) homogeneous distribution of high levels of soil nutrients (high in both proximal and distal ramets, HH); and (4) homogeneous distribution of low levels of soil nutrients (low in both proximal and distal ramets, LL). There were 16 ramet pairs for every nutrient treatment, and eight with connected stolons (C) and eight with severed stolons (S) (**Figure 1**). There were eight replicates in each of the eight treatments. The ramet pairs for the experiment were taken from eight plants and all treatments included ramet pairs originating from the same eight initial plants.

To create low level of nutrients, 0.1 g Peters Professional (20% N, 20% P, 20% K; The Scotts Company, LLC., Marysville, OH, USA) was added to the sand at the beginning of the experiment; this amount supports the survival of *A. philoxeroides* but restricts its growth. To create high level of nutrients, 0.1 g Peters Professional was added daily to each pot during the experimental period to promote plant growth. All pots were placed randomly in the greenhouse and watered daily with 100 mL of water every afternoon. The temperature in the greenhouse was about 20–35°C and the light intensity was 70% of full daylight during the experiment. The treatments were conducted for 2 months and the plants were harvested on October 15.

### Measurement and Data Analysis

Before the harvest, number of new ramets for each proximal and distal ramet was counted. The roots were then washed by hand to remove sand and plants were harvested and stored in a refrigerator (5°C) for further measurements. The total length of each stolon was measured and the plants were separated into roots, leaves, and stolons and oven-dried at 70°C for 72 h to determine the dry weight.

Before analysis, the data that did not meet normality were log transformed. We analyzed the growth and biomass allocation of *A. philoxeroides* in heterogeneous and homogeneous habitats with respect to different effects of clonal integration and various nutrient availabilities in the two habitats. The data were analyzed by using one-way analysis of variance and Duncan test. All analyses were conducted in IBM SPSS Statistics 19 (SPSS Inc., Chicago, IL, USA).

## Results

### Growth and Biomass Allocation in Heterogeneous Habitats

**FIGURE 2:**
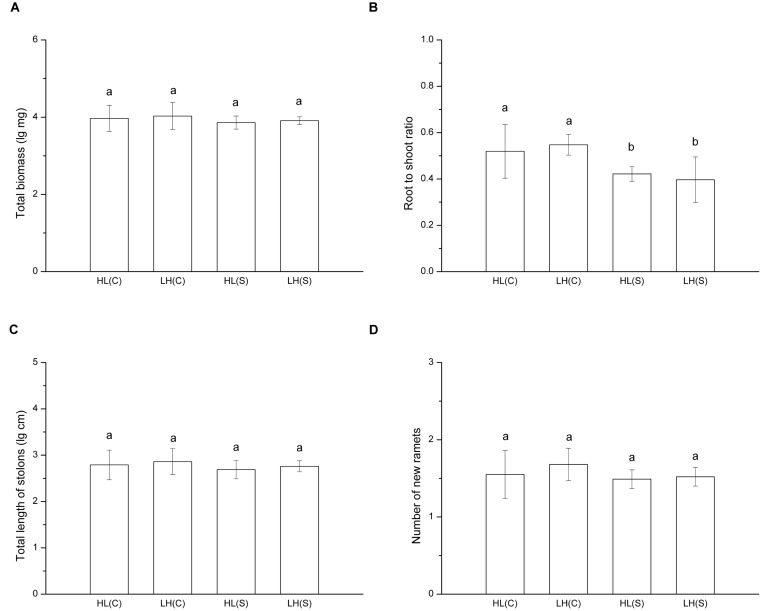
**Growth and biomass allocation of ramet pairs in heterogeneous habitats.** **(A)** Total biomass, **(B)** root to shoot ratio, **(C)** total length of stolons, **(D)** number of new ramets. Values sharing the same letter are not significantly different at α = 0.05. Total biomass, total length of stolons, and number of new ramets were log transformed to satisfy the normality and homogeneity of variance.

In treatments with heterogeneous distribution of nutrients, total biomass (*F* = 0.630, *p* > 0.05), total length of stolons (*F* = 0.699, *p* > 0.05), and number of new ramets (*F* = 1.311, *p* > 0.05) of each ramet pair (proximal ramet plus distal ramet) did not show a significant difference among treatments (**Figures 2A,C,D**). However, root to shoot ratio of ramets exposed to HL(C) and LH(C) was significantly higher than that in HL(S) and LH(S) (*F* = 6.262, *p* < 0.01; **Figure 2B**).

**FIGURE 3:**
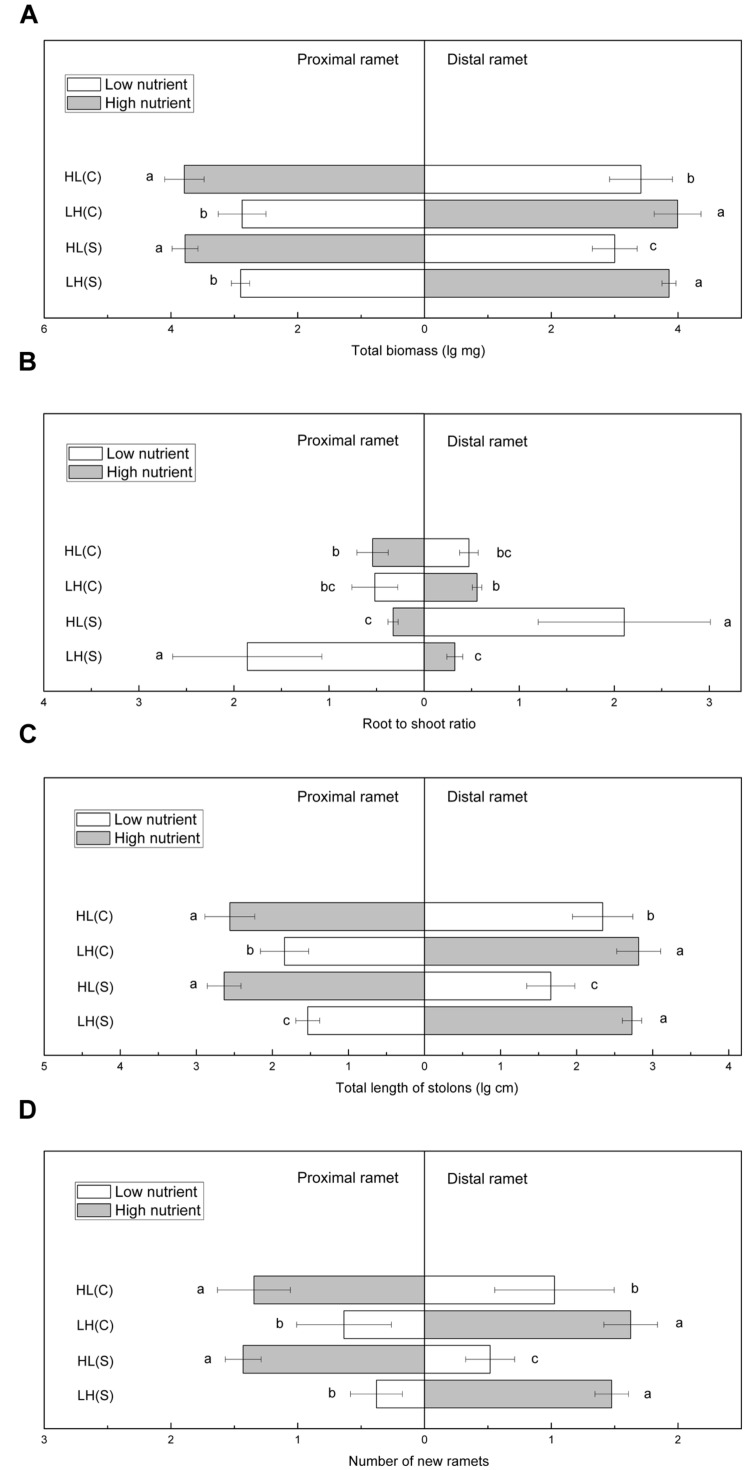
**Growth and biomass allocation of proximal and distal ramets in heterogeneous habitats.** **(A)** Total biomass, **(B)** root to shoot ratio, **(C)** total length of stolons, **(D)** number of new ramets. Values sharing the same letter are not significantly different at α = 0.05. Total biomass, total length of stolons, and number of new ramets were log transformed to satisfy the normality and homogeneity of variance.

Total biomass of proximal ramets did not differ significantly between HL(C) and HL(S), but total biomass of distal ramets was significantly higher in HL(C) than in HL(S) (**Figure 3A**). In contrast, total biomass of proximal or distal ramets did not show significant differences between LH(C) and LH(S) (**Figure 3A**). Root to shoot ratio of proximal ramets in LH(S) was the highest among the four treatments (**Figure 3B**). Root to shoot ratio of proximal ramets in HL(C) was similar to that in LH(C), but significantly higher than that in HL(S) (**Figure 3B**). Similarly, root to shoot ratio of distal ramets in HL(S) was the highest among the four treatments (**Figure 3B**). Root to shoot ratio of distal ramets in LH(C), similar to distal ramets in HL(C), exceeded that of distal ramets in LH(S) (**Figure 3B**). Total stolon length of proximal ramets in HL(C) and HL(S) was similar, whereas that of distal ramets was significantly greater in HL(C) than in HL(S) (**Figure 3C**). Likewise, total stolon length of distal ramets did not show significant difference between LH(C) and LH(S), whereas total stolon length of proximal ramets was greater in LH(C) than in LH(S) (**Figure 3C**). In addition, number of new ramets showed a similar trend to that of total biomass (**Figure 3D**).

### Growth and Biomass Allocation in Homogeneous Habitats

**FIGURE 4:**
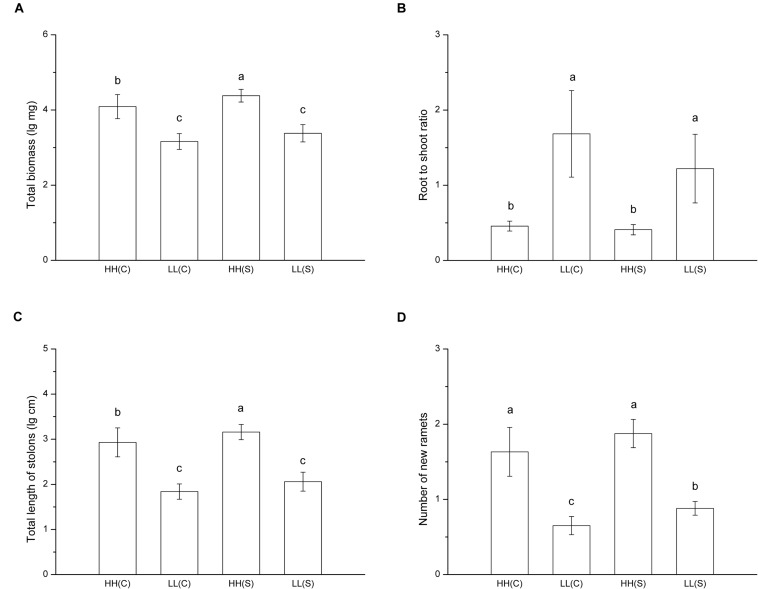
**Growth and biomass allocation of ramet pairs in homogeneous habitats.** **(A)** Total biomass, **(B)** root to shoot ratio, **(C)** total length of stolons, **(D)** number of new ramets. Values sharing the same letter are not significantly different at α = 0.05. Total biomass, total length of stolons, and number of new ramets were log transformed to satisfy the normality and homogeneity of variance.

In treatments with homogeneous nutrient availabilities, total biomass of ramet pairs (proximal ramet plus distal ramet) was significant smaller in HH(C) than in HH(S) and it did not differ between LL(C) and LL(S) (*F* = 47.927, *p* < 0.001; **Figure 4A**). Root to shoot ratio showed no difference between stolon-connected and stolon-severed treatments in high (HH[C] and HH[S]) or low (LL[C] and LL[S]) nutrient availabilities, while the ratio was much lower in high nutrient availabilities than in low nutrient availabilities (*F* = 24.673, *p* < 0.001; **Figure 4B**). Total stolon length of ramet pairs exhibited the same trends as total biomass among the four treatments (*F* = 65.073, *p* < 0.001; **Figure 4C**). Ramet pairs had similar number of new ramets between HH(C) and HH(S), but ramet pairs had less new ramets in LL(C) than in LL(S) (*F* = 26.488, *p* < 0.001; **Figure 4D**).

**FIGURE 5:**
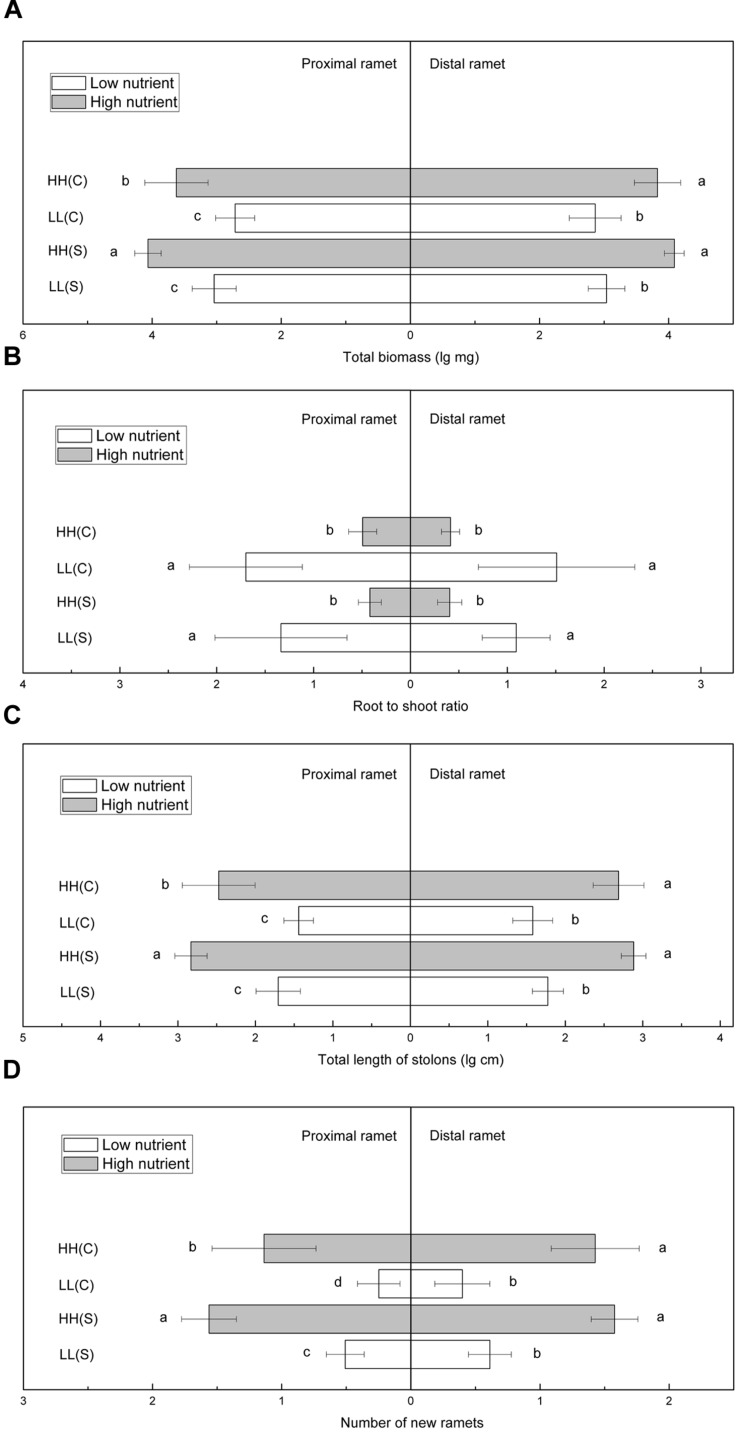
**Growth and biomass allocation of proximal and distal ramets in homogeneous habitats.** **(A)** Total biomass, **(B)** root to shoot ratio, **(C)** total length of stolons, **(D)** number of new ramets. Values sharing the same letter are not significantly different at α = 0.05. Total biomass, total length of stolons, and number of new ramets were log transformed to satisfy the normality and homogeneity of variance.

In homogeneous high nutrient conditions, total biomass of proximal ramets was significantly smaller in HH(C) than in HH(S), while total biomass of distal ramets in HH(C) and HH(S) did not show any difference (**Figure 5A**). In homogeneous low nutrient availabilities, total biomass of proximal or distal ramets showed no difference between LL(C) and in LL(S) (**Figure 5A**). Root to shoot ratio of proximal ramets was similar between HH(C) and HH(S) or between LL(C) and LL(S), and so it was for root to shoot ratio of distal ramets (**Figure 5B**). Total stolon length of proximal and distal ramets exhibited the similar trends to that of total biomass (**Figure 5C**). Number of new ramets of proximal ramets was significantly less in HH(C) than in HH(S), but that of distal ramets was not significantly different between HH(C) and HH(S) (**Figure 5D**). New ramets of proximal and distal ramets showed similar trends in low nutrient (LL[C] and LL[S]) availabilities to that in high nutrient availabilities (**Figure 5D**).

## Discussion

### Clonal Integration in Heterogeneous Habitats

In heterogeneous habitats, clonal plants translocate resources among ramets through clonal integration, promoting the growth of ramets in stressful habitats (Wang et al., 2008; Xu et al., 2010; Song et al., 2013; Roiloa et al., 2014a). In the present study, we confirmed that in heterogeneous soil nutrient conditions total biomass, total length of stolons, and number of new ramets of distal ramets were significantly greater when stolon connection between ramets were left intact than when it was severed. Clonal integration did not reduce the growth of proximal ramets, which was in agreement with previous reports (Pauliukonis and Gough, 2004; Song et al., 2013). However, clonal integration did not affect the growth of ramet pairs, disagreeing with previous findings of a meta-analysis (Song et al., 2013). This may be because performance of proximal ramets was much better than that of distal ramets in all ramet pairs under this nutrient condition.

When proximal ramets were grown in low nutrient conditions and distal ramets in high nutrient conditions, clonal integration increased total length of stolons but did not affect total biomass or number of new ramets in proximal ramets. Therefore, proximal ramets in low nutrient conditions benefited a little from distal ramets in high nutrient conditions, which agreed with the study on *Hydrocotyle peduncularis* (Peterson and Chesson, 2002). The performance of ramets in two heterogeneous habitats indicate that clonal integration of *A. philoxeroides* is bidirectional and differentiated. It is highly acropetal and lowly basipetal, and distal ramets can obtain more resources than proximal ramets when they are grown in low nutrient habitats and connected to ramets in high nutrient habitats. This effect may promote the escape of this species from barren habitats in heterogeneous nutrient environments (Zhou et al., 2012).

Clonal integration increased root to shoot ratio of ramet pairs in heterogeneous habitats. Specifically, clonal integration increased root to shoot ratio of ramets in high-nutrient conditions but decreased root to shoot ratio of ramets in low-nutrient conditions. As biomass of ramets was much larger in high-nutrient conditions than in low-nutrient conditions, root to shoot ratio of ramet pairs was dominated by ramets in the zone with high nutrient availabilities. These results indicated that labor division occurred in proximal and distal ramets in heterogeneous nutrient conditions (You et al., 2014). However, when stolon connections were severed, ramets located in low-nutrient habitats directed more biomass into roots to enhance absorption, and ramets in high-nutrient habitats allocated less biomass to roots. Therefore, *A. philoxeroides* has high plasticity in biomass allocation no matter whether stolon connection was severed or not, which may contribute to its invasiveness (Geng et al., 2007; Keser et al., 2014).

### Effects of Stolon Severing in Homogeneous Habitats

Many studies showed that stolon severing decreases the growth of distal ramets and increases the performance of proximal ramets in heterogeneous habitats (Wang et al., 2009; Song et al., 2013). It is believed that clonal integration does not affect performance of clonal plants in homogeneous habitats (Evans and Whitney, 1992; Yu et al., 2002; He et al., 2011). Nevertheless, in our study, stolon severing played significant roles in homogeneous habitats. In habitats with homogeneous distribution of high nutrient availabilities, stolon severing increased biomass of proximal ramets and the whole ramet pairs but did not affect that of distal ramets. Similarly, stolon severing increased biomass of proximal ramets and the entire clone in *Pistia stratiotes* (Wang et al., 2014). It is considered that severing eliminates the effects of distal ramets on proximal ramets (such as resource transportation, metabolic costs, and apical dominance), resulting in increasing growth of proximal ramets (Pauliukonis and Gough, 2004; Wang et al., 2009, 2014). By comparison, in habitats with homogeneous distribution of low nutrient availabilities, severing did not affect biomass of proximal or distal ramets as well as whole ramet pairs. These results demonstrated that clonal fragmentation was more advantageous than clonal integration in high nutrient habitats (Oborny and Kun, 2002).

In homogeneous habitats, root to shoot ratio was significantly lower in high-nutrient conditions than in low-nutrient conditions no matter whether stolons were severed or not. Stolon severing increased total length of stolons and number of new ramets, especially in proximal ramets, under homogeneous high-nutrient conditions, which was different from the performance of *P. stratiotes* (Wang et al., 2014). This difference is mostly the result of species-specific plasticity, just as the effects of clonal integration on morphological traits of *A. philoxeroides* and *Phyla canescens* are species-specific (Xu et al., 2012). These results also confirmed high plasticity of *A. philoxeroides*, which partly answers why *A. philoxeroides* can live in diverse habitats (Geng et al., 2007).

## Conclusion

Clonal integration, which is bidirectional and differentiated in *A. philoxeroides*, could not significantly promote biomass accumulation of ramet pairs of *A. philoxeroides* but increased total length of stolons and number of new ramets of proximal or distal ramets in stressful habitats, contributing to the spreading of *A. philoxeroides* in heterogeneous habitats. In habitats with homogeneous distribution of high nutrient availabilities, stolon severing—which often occurs due to natural and/or artificial disturbance—promoted growth of proximal ramets and ramet pairs, owing to the absence of the effects from distal ramets. Hence, *A. philoxeroides*, with high plasticity, can employ different strategies to cope with various habitats. Our study contributes to revealing roles of clonal integration in both heterogeneous and homogeneous habitats and expansion patterns of invasive clonal plants such as *A. philoxeroides* in multifarious habitats.

## Author Contributions

Conceived and designed the experiments: JL, HZ, and FL. Performed the experiments: JL and HZ. Analyzed the data: JL, HZ, and RW. Wrote the paper: HZ, JL, FL, and RW.

## Conflict of Interest Statement

The authors declare that the research was conducted in the absence of any commercial or financial relationships that could be construed as a potential conflict of interest.
